# Which are the most useful scales for predicting repeat self-harm? A systematic review evaluating risk scales using measures of diagnostic accuracy

**DOI:** 10.1136/bmjopen-2015-009297

**Published:** 2016-02-12

**Authors:** L Quinlivan, J Cooper, L Davies, K Hawton, D Gunnell, N Kapur

**Affiliations:** 1 Centre for Mental Health and Safety, University of Manchester, Manchester, UK; 2 Institute of Population Health, University of Manchester, Manchester, UK; 3 Department of Psychiatry, Centre for Suicide Research, University, Warneford Hospital, Oxford, UK; 4 School of Social and Community Medicine, University of Bristol, Bristol, UK; 5 Manchester Mental Health and Social Care Trust, Manchester, UK

**Keywords:** PSYCHIATRY, PUBLIC HEALTH, Diagnostic accuracy, HEALTH SERVICES ADMINISTRATION & MANAGEMENT

## Abstract

**Objectives:**

The aims of this review were to calculate the diagnostic accuracy statistics of risk scales following self-harm and consider which might be the most useful scales in clinical practice.

**Design:**

Systematic review.

**Methods:**

We based our search terms on those used in the systematic reviews carried out for the National Institute for Health and Care Excellence self-harm guidelines (2012) and evidence update (2013), and updated the searches through to February 2015 (CINAHL, EMBASE, MEDLINE, and PsychINFO). Methodological quality was assessed and three reviewers extracted data independently. We limited our analysis to cohort studies in adults using the outcome of repeat self-harm or attempted suicide. We calculated diagnostic accuracy statistics including measures of global accuracy. Statistical pooling was not possible due to heterogeneity.

**Results:**

The eight papers included in the final analysis varied widely according to methodological quality and the content of scales employed. Overall, sensitivity of scales ranged from 6% (95% CI 5% to 6%) to 97% (CI 95% 94% to 98%). The positive predictive value (PPV) ranged from 5% (95% CI 3% to 9%) to 84% (95% CI 80% to 87%). The diagnostic OR ranged from 1.01 (95% CI 0.434 to 2.5) to 16.3 (95%CI 12.5 to 21.4). Scales with high sensitivity tended to have low PPVs.

**Conclusions:**

It is difficult to be certain which, if any, are the most useful scales for self-harm risk assessment. No scales perform sufficiently well so as to be recommended for routine clinical use. Further robust prospective studies are warranted to evaluate risk scales following an episode of self-harm. Diagnostic accuracy statistics should be considered in relation to the specific service needs, and scales should only be used as an adjunct to assessment.

**Strengths and limitations of this study**

- We evaluated the diagnostic accuracy of widely used scales which were tested for predictive use in studies between 2002 and 2014, and included 98 600 hospital presentations of self-harm or attempted suicide.
- The study provides an important critical evaluation of the scales, including a wide range of diagnostic accuracy statistics which are likely to be useful for clinicians, commissioners and hospital risk managers.
- We did not conduct a meta-analysis due to the wide heterogeneity of the scales and studies themselves.
- We limited our analyses to cohort studies of adults which used repeat self-harm or attempted suicide as an outcome, and reported measures of diagnostic accuracy.

## Introduction

Self-harm is a frequent clinical challenge and a strong predictor of future suicide.^1^ ^2^ One in six individuals presenting to hospital with self-harm will repeat the behaviour within 1 year.^2–4^ Psychosocial assessment on presentation to hospital is a key component of recommended clinical management.^5^ ^6^ Guidelines recommend that all patients presenting to the hospital services with self-harm should receive a preliminary psychosocial assessment to determine mental capacity and evaluate willingness to stay for further treatment.^5^ Mental health professionals should conduct a more comprehensive evaluation of risk and needs at a later stage, and risk scales are typically a core component of assessments despite limited evidence of their effectiveness.^6^ ^7^ Some clinical guidelines advise against the use of scales to determine management, but suggest they can be used to help structure assessments.^6^ Other guidelines recommend that only scales that have undergone formal testing should be used as part of clinical assessments.^8^

Our recent study in 32 English hospitals found that at least 20 risk tools were in use, suggesting that there is a lack of consensus over which scales are best for evaluating risk of further self-harm.^7^ The uncertainty is perhaps due to methodological differences between studies and variable standards of reporting. There are a small number of reviews which consider the predictive ability of risk scales for repeat self-harm which may help clinicians to select the most helpful tools^6^ ^9^ ^10^ but the information provided is mostly limited to dual indicators such as sensitivity/specificity, positive/negative predictive values, and there is little practical guidance for clinicians in selecting the ‘most useful tools’.

While these dual indicators are useful for determining the predictive validity of a scale, a broader range of diagnostic test criteria may be helpful when selecting an appropriate scale for clinical use given the inevitable trade-off between sensitivity (the proportion of individuals who repeat self-harm identified by the test as high risk) and specificity (proportion of people who did not repeat self-harm identified as low risk by the test). For example, a highly sensitive test might identify all patients at risk of future self-harm but could be over inclusive with cost and resource implications. Conversely, the higher threshold inherent in highly specific tests may result in false negatives and a host of deleterious consequences for patients and clinical services.

We have conducted a systematic review of existing research on risk scales to consider these issues.

The objectives were to:

1. Investigate the performance of risk scales following self-harm or attempted suicide on a wide range of dual measures, as well as more global measures of accuracy.
2. Consider which might be the most useful scales following self-harm in clinical practice settings.

This information may be useful to clinicians, commissioners and hospital risk managers, who need to critically evaluate scales for use in clinical practice.

## Method

This study extends the reviews carried out as part of the National Institute for Health and Care Excellence (NICE) self-harm guidelines^6^ and evidence update^11^ on the use of risk scales for repeat self-harm. We included recent evidence and considered a much broader range of diagnostic accuracy statistics than the original reviews.

### Literature search

We identified studies evaluating the predictive validity of risk scales for repeat self-harm from the NICE review on the longer term management of self-harm and the evidence update.^6^ ^11^ We used the same published search strategy^11^ (see online supplementary appendix 1) on CINAHL, EMBASE, MEDLINE and PsychINFO databases through to February 2015. Reference lists were also screened and related references reviewed.

### Inclusion and exclusion criteria

Consistent with the NICE self-harm evidence update,^11^ studies were included if they used a cohort design—the optimal design for evaluating the diagnostic accuracy of scales as case–control studies can overestimate diagnostic accuracy.^12^ Although suicide is an extremely important outcome following self-harm, the low base-rate hinders predictive efforts even in high-risk populations.^13^ We focused on repeat self-harm or attempted suicide as an outcome, as the incidence rate is higher and the prediction of repetition may more feasible than predicting suicide.^14^ Studies were included if measures of diagnostic accuracy (such as sensitivity, specificity and positive predictive values) were reported.

Studies were excluded if the scales were validated on a specific or restricted samples (eg, veterans, prisoners or specialist mental healthcare population), or a sample which did not include people presenting with self-harm or attempted suicide. One study^15^ recruited a mixed sample of people (presenting with suicide ideation or self-harm), but since a majority of the sample (>75%) had a history of self-harm and the study outcome was self-harm repetition, this study was included.

Some tools were validated in more than one setting and these were included once in the final analysis, using the original paper, if this met the inclusion criteria. We did this in order to gain an indication of the ‘best-case’ scenario for different instruments (the first study of a new screening tool in a setting where it was developed might be expected to give the most positive results) and because of the potential difficulty of combining measures of diagnostic accuracy from different settings. However, in order to contextualise results we did also examine the broader performance of scales which had been tested in multiple studies in a post hoc analysis.

### Assessment of bias and study quality

Study bias was evaluated at the study level using the QUADAS (Quality Assessment of Diagnostic Accuracy Studies) and STARD (Standards for Reporting of Diagnostic Accuracy) guidelines.^16^ ^17^

### Statistical analysis

True positives, false positives, true negatives and false negatives were extracted from the papers by two researchers (LQ and JC) independently, and results discussed with the third author (NK). Authors were contacted where these data were unavailable.

We used a wide range of recommended diagnostic accuracy estimates^18^ ^19^ to evaluate the predictive validity of the risk scales (box 1 and see online supplementary appendix 2), including sensitivity (proportion of individuals who repeat self-harm identified as high risk by the test); specificity (proportion of people who did not repeat self-harm identified as low risk by the test); positive predictive values (probability that a person identified by the test as high risk will actually go onto self-harm); negative predictive values (probability that a person identified as low risk will not go onto self-harm).

Box 1 **Measures of diagnostic accuracy for scales following self-harm**

Key terms

- Sensitivity (Sens): Proportion of individuals who repeat self-harm identified as high risk by the test, that is, how well the test identifies patients who repeat self-harm
- Specificity (Spec): Proportion of people who did not repeat self-harm identified as low risk by the test, that is, how well the test identifies patients who will not repeat self-harm
- Positive predictive value (PPV): The probability that the person identified as at risk for repeat self-harm will actually repeat self-harm
- Negative predictive value (NPV): The probability that the person identified as low risk for repeat self-harm will not actually repeat self-harm
- Positive likelihood ratio (LR+): How much more likely a positive test result is to occur in a patient who repeats self-harm versus one who does not repeat
- Negative likelihood ratio (LR−): How much less likely a negative test result is to occur in a patient who repeats self-harm compared to a patient who does not repeat
- Diagnostic OR (DOR): Overall global measure of test performance and represents the strength of the association between the test result and repeat self-harm (interpreted the same as an OR)
- Number allowed to diagnosis (NAD): The number of people correctly classified as having a repeat self-harm episode before an misclassification occurs^20^

Positive and negative likelihood ratios (how much more or less likely test results are to occur in patients who repeat self-harm vs those who do not) were also calculated.^19^ Likelihood ratios of 1 indicate no change in likelihood of disease or outcome (in this case repeat self-harm). Positive likelihood ratios between 1–2, 2–5, 5–10 and >10 indicate minimal, small, moderate and large increases in risk, respectively.^19^ ^21^ Negative likelihood ratios of 0.5–1.0, 0.02–0.5, 0.1–0.2 and <0.1 indicate minimal, small, moderate and large decreases in risk.^21^

We also calculated global diagnostic statistics that summarise the diagnostic performance of a test as a single indicator,^18^ including the ‘number allowed to diagnose’ (number of individuals who are correctly assigned as at high risk of repetition before one is misassigned),^20^ and the diagnostic OR^18^ (odds of positivity in repeater relative to the odds of non-repeater). Higher values indicate greater test discriminatory power.^18^ ^20^

CIs for sensitivity and specificity were calculated using the Wilson score method without correction.^22^ CIs for positive and negative likelihood ratios were produced using the method of Simel *et al*.^23^ The CI for the diagnostic OR was produced using the method published by Armitage and Berry.^24^ CIs for ‘number allowed to diagnose’ were constructed using the method based on constant χ^2^ boundaries from Press *et al*.^25^ Results are unpooled due to heterogeneity in the studies.

STATA V.13.0; StataCorp. Stata Statistical Software: Release 13. College Station, Texas: StataCorp LP, 2013) and RevMan V.5.1 (Cochrane Collaboration)^26^ were used for statistical analysis.

## Results

### Search results

**Figure 1:**
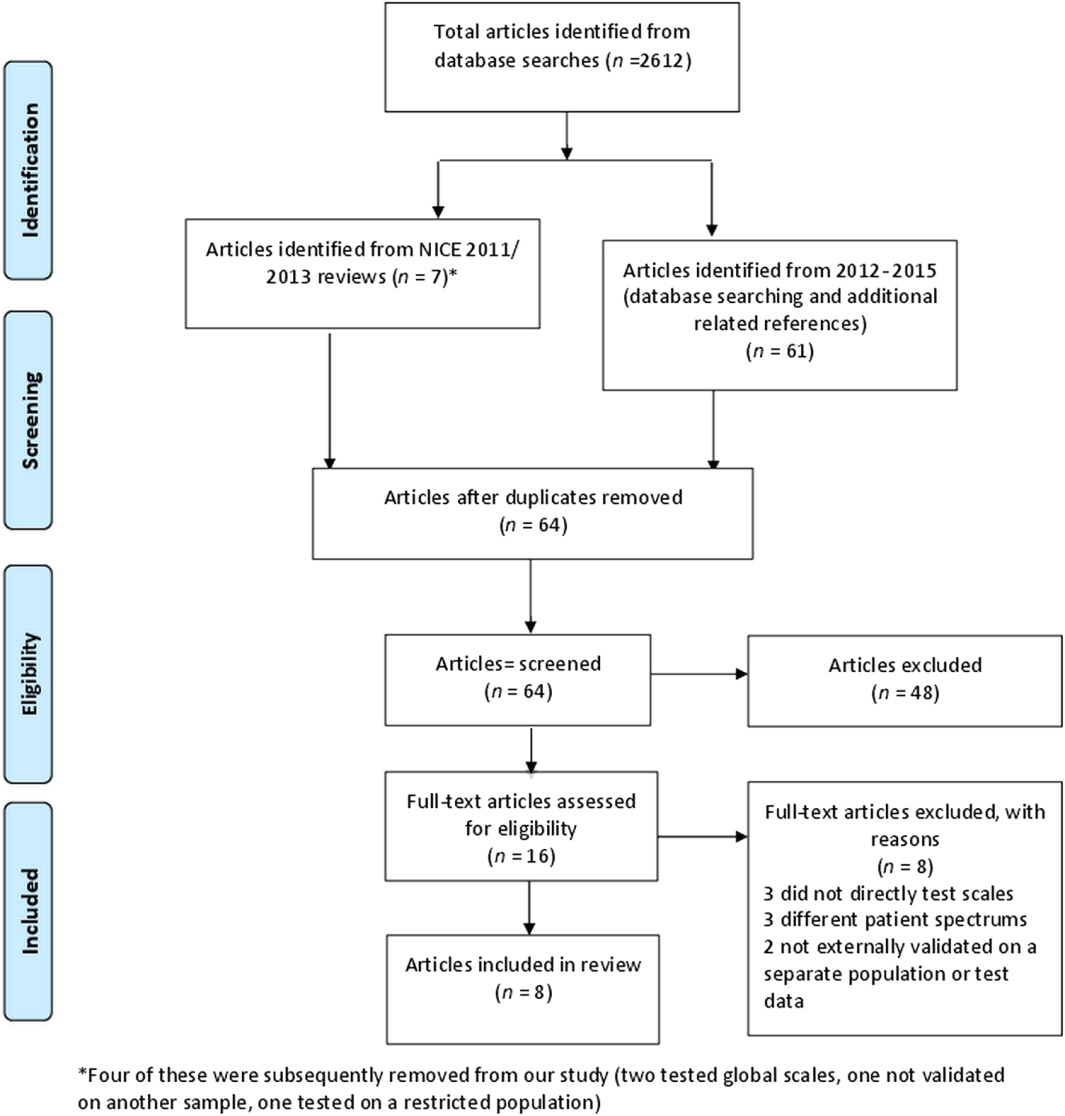
Preferred Reporting for Systematic Reviews and Meta-Analyses flow diagram^17^ describing the search process for included studies. NICE, National Institute for Health and Care Excellence.

The NICE 2011 review on the longer term management of self-harm included seven cohort studies testing the predictive validity of risk scales for repeat self-harm.^27–33^ Four were excluded as they did not meet our inclusion criteria—they examined global measures rather than scales^28^ ^32^—were statistically derived without testing in a defined cohort,^30^ or used a restricted clinical population^31^ (figure 1). The NICE evidence update^11^ included one additional cohort study.^34^ The search strategy from January 2012 to February 2015 resulted in an additional 60 papers of which three were relevant prospective cohort studies,^15^ ^35^ ^36^ and one additional cohort study^14^ was retrieved from related references (see figure 1). We also reran the searches for the earlier time periods. No additional studies were identified. In total, there were eight studies examining 11 scales which were included in the final analysis (figure 1).

### Description of studies

The methodological characteristics of the eight studies evaluating 11 scales are described in table 1. Further detailed information on bias and reporting is presented in online supplementary appendix 3. The studies were conducted between 2002 and 2014, and included 98 600 hospital presentations of self-harm or attempted suicide. In terms of service context, the studies were generally carried out across multiple sites, the majority in publicly funded health services. Four studies were based on self-harm emergency department populations.^15^ ^27^ ^35^ ^36^ Randall *et al*^15^ included a mix of patients presenting with self-harm or suicidal ideation. One study was based on patients treated for self-poisoning.^29^ Two studies were based on hospital presentations for suicide attempts with suicidal intent as an inclusion criterion,^33^ ^34^ and one study^14^ was based on patients admitted to a medical bed after self-harm. The length of follow-up ranged between 3 and 36 months, and outcome data was mostly ascertained through hospital databases. The incidence of repeat self-harm across studies ranged from 3%^34^ to 37%,^33^ possibly suggesting differences in casemix.

**Table 1:** Methodological characteristics of the studies

Study ID	Index test and comparator tests	Participants	Outcome events	Sampling and clinical population	How assessment was conducted	Context	Outcomes	Reference standard	Follow-up (months)	Estimates of 95% CI for diagnostic accuracy	Included raw data
Cooper *et al*^27^ (England)	MSHR (development and validation)	2095	373	Consecutive emergency department self-harm presentations	Variables gathered as part of routine assessment and extracted from a database	Multisite, within publically funded National Health Service	Self-harm and suicide	Hospital database records, searched by definition	6	Yes	Yes
Steeg *et al*^36^ (England)	ReACT rule (development and validation)	7039	2096	Consecutive assessed and non-assessed emergency department self-harm presentations (England)	Variables gathered as part of routine assessment and extracted from a database	Multisite, within publically funded National Health Service	Repeat self-harm and suicide	Hospital database records, searched by definition	6	Yes	No
Bilèn *et al*^35^ (Sweden)	SSHR, MSHR	325	80	Consecutive emergency department self-harm presentations	Scales completed by treating physician	Two large university hospitals with emergency departments, within publically funded National Health Service	Repeat self-harm	Hospital database records, searched by definition	6	Yes	Yes
Spittal *et al*^14^ (Australia)	RESH (development and validation)	84 659	21 672	Consecutive inpatients admitted for self-harm	Large linked data gathered as part of hospital admissions for self-harm and suicide. Study variables extracted from database	Multisite, private and publically funded hospitals	Repeat self-harm and suicide combined	Hospital database records, searched by definition	6	Yes	No
Carter *et al*^29^ (Australia)	ERRS	1317	188	Consecutive self-poisoning patients presenting for hospital treatment at centralised referral centre	Data gathered by toxicology and psychiatric staff and rated by psychiatric staff (psychiatrist, psychiatric registrars, clinical nurse consultants) rated ERRS variables based on clinical interviews, patient self-report, and case notes	Tertiary specialist service for self-poisoning	Repeat self-poisoning	Hospital database records, searched by definition	12	No	No
Randall *et al*^15^ (Canada)	GSI, BIS DAST BHI, CAGE, MSHR	157	34	Emergency department presentations with self-harm or suicidal ideation	Trained researcher administered standardised interview and conducted chart reviews	Two teaching hospitals with largest emergency departments in Edmonton	Repeat self-harm	Hospital records and telephone call	3	Yes for ROC	No
Waern^33^ (Sweden)	SUAS	162	56	Unclear sampling, patients admitted to ED wards after a suicide attempt with at least some intent to die	Face-to-face interviews carried out by three psychiatric nurses and one psychiatrist within 3 days of attempt	Publically funded University hospital, which is the only hospital to provide emergency services in the study area	Repeat suicide attempts and suicide	Hospital database records, search strategy unclear	36	No	No
Bolton *et al*^34^ (Canada)	SPS, MSPS	2846	80	Consecutive adult referrals to psychiatric services from the emergency department Based on C-CASA, 2 groups established: suicide attempts defined with intent and a reference group without any suicidal ideation, behaviour, or preparatory acts towards suicide attempts	Scales completed by psychiatric residents under supervision by attending psychiatrist, subsequent to assessment	Two largest tertiary care teaching hospitals in Manitoba	Future suicide attempts	Unclear	6	No	Yes

BHI, Beck Hopelessness Scale; BIS, Barratt Impulsivity Scale; C-CASA, Columbia Classification Algorithm of Suicide Assessment; CAGE, Cut down, Annoyed, Guilt, Eye-opener; DAST, Drug Abuse Screening Test; ED, emergency department; ERRS, Edinburgh Risk of Repetition; GSI, Global Severity Index; MSHR, Manchester Self-Harm Rule; MSPS, Modified SAD PERSONS Scale; ReACT, ReACT Self-Harm Rule; RESH, Repeated Episodes of Self-Ham score; ROC, receiver operating characteristic; SPS, SAD PERSONS Scale; SSHR, Söderjukuset Self-harm Rule; SUAS, Suicide Assessment Scale.

Four studies involved developing a tool which was then validated on a split site or external data set.^14^ ^27^ ^35^ ^36^ The remainder were validation studies of existing scales.^15^ ^29^ ^33^ ^34^ The scales varied in length ranging from four items (Manchester Self-harm Rule, ReACT Self-Harm Rule 37) to 53 items for the Global Severity Scale. Most scales included previous history of self-harm or suicide attempts or prior psychiatric treatment as items. Others scales items included personality factors (Barratt Impulsivity Scale, clinical symptomology (eg, Global Severity Index), drug misuse (eg, Drug Abuse Screening Test) and variations in symptoms associated with suicidal thoughts and behaviours (eg, Suicide Assessment Scale).

None of the studies were explicitly formatted according to standard guidelines (eg, STARD^17^) and reporting varied across the studies. For example, there were variations across studies in the reporting of recruitment flow^34^ and patient characteristics,^29^ ^33^ ^34^ cross-tabulations of raw data,^14^ ^33^ ^34^ ^36^ CIs for diagnostic accuracy statistics,,^15^ ^29^ ^33^ and use of thresholds (eg, Randall *et al*^15^ did not use any). The database studies^14^ ^27^ ^35^ ^36^ were the most robustly reported according to STARD indices.

### Diagnostic accuracy statistics

**Figure 2:**
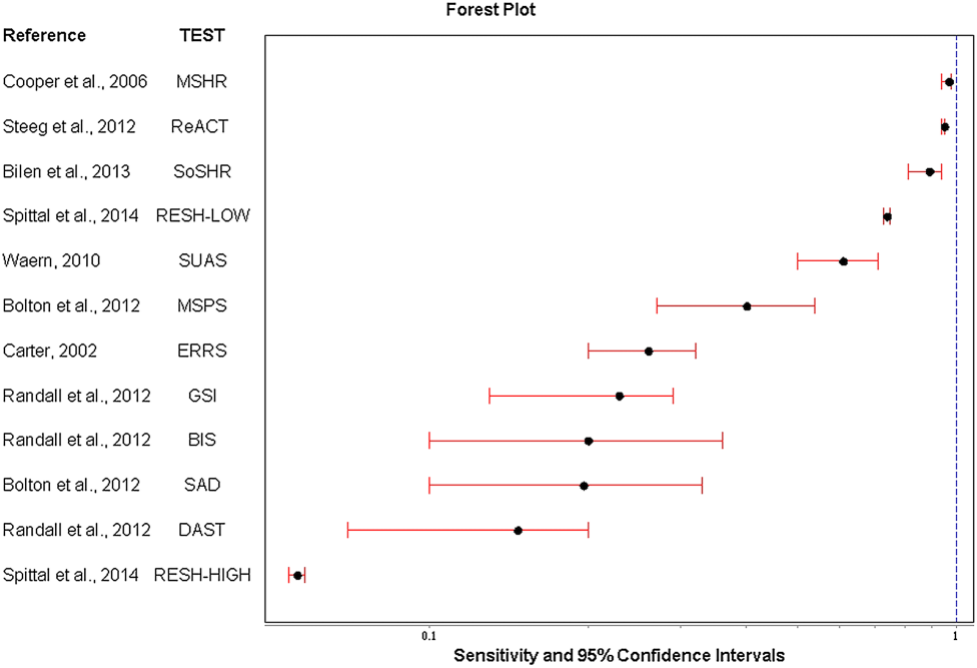
Forest plot of sensitivity and 95% CIs for individual scales. BIS, Barratt Impulsivity Scale; DAST, Drug Abuse Screening Test; ERRS, Edinburgh Risk of Repetition; GSI, Global Severity Index; MSHR, Manchester Self-Harm Rule; MSPS, Modified SAD PERSONS Scale; ReACT, ReACT Self-Harm Rule; RESH, Repeated Episodes of Self-Ham score; SoSHR, Söderjukuset Self-harm Rule; SUAS, Suicide Assessment Scale.

**Figure 3:**
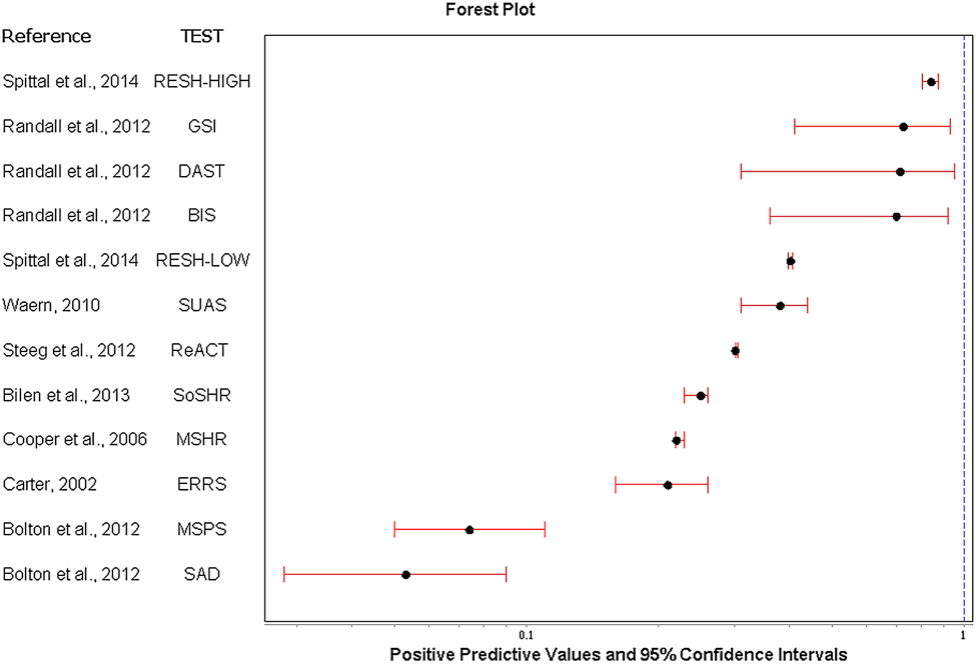
Forest plot of positive predictive values and 95% CIs for individual scale. BIS, Barratt Impulsivity Scale; DAST, Drug Abuse Screening Test; ERRS, Edinburgh Risk of Repetition; GSI, Global Severity Index; MSHR, Manchester Self-Harm Rule; MSPS, Modified SAD PERSONS Scale; ReACT, ReACT Self-Harm Rule; RESH, Repeated Episodes of Self-Ham score; SoSHR, Söderjukuset Self-harm Rule; SUAS, Suicide Assessment Scale.

The full range of diagnostic accuracy statistics are presented in table 2. Figures 2 and 3 show forest plots for sensitivity and positive predictive values, respectively. Sensitivity (how well the test identifies people who repeat self-harm) ranged from 5.6% for the Repeated Episodes of Self-Harm scale^14^ using the threshold for the highest risk to 97% for the Manchester self-harm rule^27^ 95% for the ReACT Self-Harm rule,^36^ and 89% for the Söderjukuest Self-harm Rule.^35^

**Table 2:** Diagnostic accuracy statistics with 95% CIs*

Scale	Reference	Prevalence	Sensitivity	Specificity	PPV	NPV	LR+	LR−	NAD	DOR
BIS	Randall *et al*^15^	0.22	0.20 (0.10 to 0.36)	0.97 (0.94 to 0.99)	0.70 (0.36 to 0.92)	0.78 (0.75 to 0.80)	6.8 (1.67 to 32.40)	0.82 (0.74 to 0.96)	4.4 (3.6 to 5.1)	8.25† (1.76 to 43.6)
DAST	Randall *et al*^15^	0.22	0.15 (0.07 to 0.20)	0.98 (0.95 to 0.99)	0.71 (0.31 to 0.95)	0.78 (0.75 to 0.79)	7.5 (1.4 to 55.1)	0.87 (0.80 to 0.98)	4.4 (3.7 to 4.9)	8.66† (1.37 to 68.69)
ERRS	Carter *et al*^29^	0.14	0.26 (0.20 to 0.32)	0.84 (0.83 to 0.85)	0.21 (0.16 to 0.26)	0.88 (0.87 to 0.90)	1.63 (1.21 to 2.16)	0.88 (0.80 to 0.96)	4.2 (3.9 to 4.5)	1.85 (1.26 to 2.71)
GSI	Randall *et al*^15^	0.22	0.23 (0.13 to 0.29)	0.97 (0.93 to 0.99)	0.73 (0.41 to 0.93)	0.78 (0.75 to 0.80)	7.54 (1.94 to 35.0)	0.79 (0.71 0.94)	4.5 (3.6 to 5.2)	9.48† (2.07 to 48.95)
MSPS	Bolton *et al*^34^	0.03	0.40 (0.27 to 0.54)	0.85 (0.85 to 0.86)	0.07 (0.05 to 0.11)	0.98 (0.98 to 0.99)	2.73 (1.83 to 3.76)	0.70 (0.54 to 0.85)	6.3 (6.0 to 6.6)	3.87 (2.15 to 6.96)
MSHR	Cooper *et al*^27^	0.17	0.97 (0.94 to 0.98)	0.26 (0.26 to 0.27)	0.22 (0.22 to 0.23)	.097 (0.96 to 0.99)	1.31 (1.27 to 1.34)	0.12 (0.07 to 0.22)	1.6 (1.6 to 1.7)	10.77 (6.00 to 20.3)
ReACT	Steeg *et al*^36^	0.30	0.95 (0.94 to 0.95)	0.21 (0.21 to 0.21)	0.30 (0.30 to 0.31)	0.91 (0.90 to 0.92)	1.19 (1.18 to 1.20)	0.26 (0.23 to 0.29)	1.7 (1.7 to 1.7)	4.58 (4.07 to 5.18)
SAS	Waern *et al*^33^	0.37	0.61 (0.50 to 0.71)	0.40 (0.33 to 0.50)	0.38 (0.31 to 0.44)	0.63 (0.50 to 0.72)	1.00* (0.75 to 1.30)	0.99* (0.64 to 1.5)	1.9 (1.7 to 2.2)	1.01 (0.50 to 2.04)‡
SPS	Bolton e*t al*^34^	0.03	0.20 (0.10 to 0.33)	0.91 (0.91 to 0.91)	0.05 (0.03 to 0.09)	0.98 (0.97 to 0.98)	2.11 (1.10 to 3.71)	0.89 (0.74 to 0.99)	9.0 (8.6 to 9.6)	2.38 (1.10 to 5.04)
SSHR	Bilén *et al*^35^	0.24	0.89 (0.81 to 0.94)	0.11 (0.09 to 0.13)	0.25 (0.23 to 0.26)	0.76 (0.60 to 0.88)	1.00† (0.90 to 1.10)	0.98† (0.44 to 2.1)	1.4 (1.4 to 1.5)	1.01 (0.434 to 2.5)‡
RESH LOW	Spittal *et al*^14^	0.26	0.74 (0.73 to 0.75)	0.63 (0.62 to 0.63)	0.40 (0.40 to 0.41)	0.88 (0.87 to 0.88)	1.97 (1.93 to 2.0)	0.42 (0.40 to 0.44)	2.8 (2.8 to 2.9)	4.72 (4.42 to 5.03)
RESH HIGH	Spittal *et al*^14^	0.26	0.06 (0.05 to 0.06)	0.996 (0.995 to 0.997)	0.84 (0.80 to 0.87)	0.76 (0.76 to 0.76)	15.74 (11.88 to 20.22)	0.95 (0.95 to 0.95)	4.1 (4.1 to 4.14)	16.34 (12.49 to 21.39)

*Numbers may be different from reported in original studies as the statistics were calculated using raw data where possible.

†Wide CIs due to smaller sample size.

‡Overlapping CI indicating no effect.

BIS, Barratt Impulsivity Scale; DAST, Drug Abuse Screening Test; DOR, diagnostic OR; ERRS, Edinburgh Risk of Repetition; GSI, Global Severity Index; LR+, positive likelihood ratio; LR−, negative likelihood ratio; MSHR, Manchester Self-Harm Rule; MSPS, Modified SAD PERSONS Scale; NAD, number allowed to diagnosis; NPV, negative predictive value; PPV, positive predictive value; ReACT, ReACT Self-Harm Rule; RESH, Repeated Episodes of Self-Ham score; SPS, SAD PERSONS Scale; SSHR, Söderjukuset Self-harm Rule.

Positive predictive values for the latter high sensitivity scales were low (26%, 21% and 11%, respectively) and were highest for the Repeated Episodes of Self-Harm scale at the highest threshold (84%)^14^ followed by the Global Severity Index (73%),^15^ and the Drug Abuse Screening Test^15^ (figure 3). It should be noted that the Repeated Episodes of Self-Harm score was tested on inpatients admitted to hospital services for self-harm.^14^

Positive likelihood ratios ranged from 15.7 for the Repeated Episodes of Self-Harm scale^14^ at the highest threshold (indicating a large increase in the likelihood of repetition) to 1.0 for Söderjukuset Self-harm Rule^35^ and the Suicide Assessment Scale^33^ (indicating no change in the likelihood of repetition) (table 2). The diagnostic OR which presents the accuracy of a test as a global single indicator ranged from 16.34 (Repeated Episodes of Self-Harm scale at the highest threshold^14^) and 10.77 (Manchester Self-Harm Rule^27^) to 1.01 for the Södersjukuset Self-harm Rule^35^ and the Suicide Assessment Scale^33^ (table 2).

Although the length of follow-up varied, there were no clear patterns in relation to the prediction of shorter versus longer term risk. As noted previously, there was a wide variation in the methodological characteristics of the studies and in the scales themselves.

### Operational issues

Operational characteristics (ie, the time taken to do the scale, technical specifications, ease of use, cost, staff training, user acceptability) are important to the clinical use of a scale and are listed in detail in table 3. Scales with characteristics which may need to be considered before their use include the Global Severity Index (copyright protected, costs associated with use, a 53-item scale with training required prior to use).^37^ The Drug Abuse Screening test may also be limited for clinicians working with self-harm populations, as the test is designed to assess drug-related problems.

**Table 3:** Scale operational factors

Instrument	Purpose of instrument	Cost	Description	Time (min)	Copyright	Administration	Training	Study reference	Original scale reference
The Manchester Self-Harm Rule	Risk-stratification model for use with ED staff in the assessment of self-harm to discriminate between patients and higher vs lower risk of repetition or subsequent suicide by 6 months	Free	4 screening items, dichotomous answers (history of self-harm, prior psychiatric treatment, benzodiazepine overdose, current psychiatric treatment) (1=present, 0=absent), positive answer is a positive result	5	No	Paper and pen	Limited training necessary	Cooper *et al*^27^	Cooper *et al*^27^
The ReACT Self-Harm Rule	Screening tool to identify patients at higher risk of repeat self-harm suicide within 6 months of ED self-harm presentation	Free	4 items (recent self-harm (in the past year), Alone of homeless (living status), cutting used as a method of harm, and treatment for a current psychiatric disorder), presence of one or more of these items classifies patient as at higher risk of repeat self-harm/suicide within 6 months	5	No	Paper and pen	Limited training necessary	Steeg *et al*^36^	Steeg *et al*^36^
The RESH Self-Harm tool	Designed to assist clinicians in clinical management of self-harm patients	Free	4 main items with an assigned weight: number of prior episodes (0, 1, 2, 3, 4, 5, 6 or more), time between episodes (1–60 days, 61 days to 12 months, > 12 months), psychiatric diagnosis in the last 12 months (substance misuse disorder, depression, anxiety, eating disorder, personality disorder), and psychiatric stay in the last 12 months. The RESH scale was constructed using a weighted scoring algorithm based on the log ORs based on 0 to 20. It has five cut-off points ranging from low-risk to high-risk that can be applied to different interventions	Unknown	No	Paper and Pen	Unknown	Spittal *et al*^14^	Spittal *et al*^14^
The Global Severity Index (GSI)	The GSI symptom scale is a component of the Brief Symptom Inventory (BSI). Also a global indicator, the Symptom Checklist-90-Revised (SCL-90-R) is ‘designed to help quantify a patients severity-of-illness and provides a single composite score for measuring the outcome of a treatment programme based on reducing symptom severity’ (Pearson Assessments)	£118.32 per 50 answer sheets with test items, 50 profile forms and 2 worksheets (£935+vat for 500)	53-item self-report on 5-point rating scale. 9 symptom dimensions (somatisation, Obsessive-compulsive, Interpersonal sensitivity, Depression, Anxiety, Hostility, Phobic Anxiety, Paranoid Ideation, Psychoticism) Global Indices (Global severity index, Positive Symptom Distress Index, Positive Symptom total). Also a component of the 90-item SCL-90-R (12–15 min to complete)	8–10	Pearson Assessments	Q Local Software, Mail-in scoring service, Hand scoring, or optimal Scan Scoring	B, Q1, Q2 level	Randall *et al*^15^	Derogitis and Melisaratos 37
The SAD PERSONS scale	Educational tool for medical students to determine suicide risk	Free	10-item mnemonic consisting of risk factors based on literature review (male sex, age, depression, previous attempt, excess alcohol or substance abuse, rational thinking loss, social supports lacking, organised plan, no spouse, sickness). Items scored 1 if present, 0 if absent. Cut-off points: 3 categories of suicide risk, low, moderate, and high (0–4, 5–6 and 7–10, respectively)	5–10	No	Paper and pen	Limited training necessary	Bolton *et al*^34^	Patterson *et al*^38^
The Modified SAD PERSONS	Suicide assessment in the ED		10-item scale. Modified SAD PERSONS by adding five additional criteria (feelings of hopelessness, history of psychiatric care, drug addiction, a ‘serious’ attempt, and affirmative or ambivalent answers when questioned about future intent regarding suicide. Four scale items are weighted with scores of 2 to give a total possible score of 14. Cut-off points: low (0–5), moderate (6–8), and high (9–14)	5–10	No	Paper and pen	Limited training necessary	Bolton *et al*^34^	Hockberger and Rothstein^39^
The Barratt Impulsivity Scale	Designed to assess the personality trait of impulsiveness	Free	30 items based on personality. Self-report. Responses scored on a 4-point likert scale. Responses summed to total score	10	No	Paper and pen	None	Randall *et al*^15^	Patton *et al*^40^
The Drug Abuse Screening Test	Designed to identify patients who are abusing drugs, also to provide a quantitative index score if the degree of problems related to drug use and misuse	Free	28-items self-report Responses are dichotomous (1=yes, 0=no), except for items 4, 5, and 7 which are reverse coded. The scale is unidimensional with a total score calculated from summing all the items that are positive in relation to increased drug use. Cut-off scores of six to 11 are used for identifying patients with drug abuse/ misuse problems, whereas of 16 or above is considered as severe drug abuse or dependency	5	No	Paper and Pen	Adherence to instructions for administration and scoring provided with the scale	Randall *et al*^15^	Skinner^41^
The Suicide Assessment Scale	Designed to be sensitivity to change in suicidality over time and in treatment	Free	20 items, on a 0–4 likert scale summed to arrive at a maximum score of 80. Five main areas covered: (affect (5 items), bodily states (5 items), control and coping (5 items), emotional reactivity, suicidal thoughts and behaviour (5 items). Clinician and self-report versions available	>30=high risk	No	Paper and pen	Instructions available	Waern *et al*^33^	Stanley *et al*^42^ Niméus *et al*^43^
Edinburgh Risk of Repetition Scale	Designed to identify patients at risk of repeat self-harm	Free	11 items (previous self-harm, personality disorder, alcohol, previous psychiatric care, unemployment, social class, drug abuse, criminal record, violence, age (25–54), civil status (single, divorced, separated). Each positive item, scored as 1, total ranges from 0–11	Men >8=high risk Women >6=high risk	No	Paper and Pen	None	Carter *et al*^29^	Kreitman and Foster^44^
Söderjukhuset Self-Harm Rule	Designed to identify patients at risk of repeat self-harm	Free	4 items (gender, current psychiatric treatment, previous self-harm, antidepressant treatment)	Regression model: risk above 0.14 predicted to be repeater	No	Paper and pen and calculator	None, but familiarity with regression models and calculation necessary	Bilén *et al*^35^	Bilén *et al*^35^

ED, emergency department; RESH, Repeated Episodes of Self-Ham score.

## Discussion

### Main findings

Risk scales are in widespread use in health services managing self-harm patients.^7^ We examined the diagnostic accuracy of a number of scales after self-harm and found a wide variation in samples, follow-up, reporting, thresholds, as well as differences in the content of the scales themselves. This heterogeneity was reflected in the variation in predictive accuracy across scales. For example, the Manchester Self-Harm Rule was high in sensitivity (97%) but had low positive predictive value (22%).^27^ Conversely, the Drug Abuse Screening Test had low sensitivity (15%) but high positive predictive value (98%).^15^ Scales which scored highly on global measures of diagnostic accuracy included the Repeated Episodes of Self-Harm scale at the highest threshold^14^ (16.34), the Manchester Self-Harm Rule^27^ (10.77), the Drug Abuse Screening Test^15^ (8.66) and the Barratt Impulsivity Scale^15^ (8.25), but even these scales varied markedly in their sensitivity from 6% for the Repeated Episodes of Self-Harm scale^14^ to 97% for the Manchester Self-Harm Scale.^27^

### Methodological limitations

We did not conduct any meta-analyses due to the heterogeneity of the studies, nor did we calculate the receiver operating characteristics of the scales as we did not have the raw interval data. However, we provided a range of diagnostic accuracy statistics and associated CIs, which are useful in the critical evaluation of risk scales following self-harm. Some scales were tested in several settings, and we made no attempt to pool accuracy statistics across studies. Instead, we focused on a single study for each scale. This was the original study where this met inclusion criteria. We did this in order to gain an indication of the scale performance under potentially optimal conditions and because of the difficulty in pooling results from different settings.

Two scales in particular had been tested in multiple studies and settings (Edinburgh Risk of Repetition Scale and the Manchester Self-Harm Rule).^9^ Sensitivities for the Edinburgh Risk of Repetition Scale ranged from 26% to 41%, and specificities ranged from 84% to 91% in an early study.^44^ A further validation study conducted in Australia provided similar results (sensitivity: 26%, specificity: 84%).^29^ Broadly similar results were found in Oxford,^45^ for the Edinburgh Risk of Repetition Scale, but sensitivities were lower when tested on a 12-month rather than a 6-month follow-up, and ranged from 3% to 16%.^45^

The Manchester Self-Harm Rule was validated in Sweden,^35^ Manchester^28^ ^36^ and Canada.^15^ The results were similar to those of Cooper *et al*^27^ in demonstrating the high sensitivity (94%, 94%, 98% and 95.1% for the studies, respectively) and low specificity (18%, 26%, 17% and 14.7%, respectively) of the scale.

We were keen to replicate the searches carried out as part of UK national guidance as far as possible. In some senses, the current paper was intended as an update of the review carried out as part of the NICE self-harm (longer term management guidelines), and we were constrained by the original methodology. Some well-known scales were not included in the NICE review^6^ ^11^ on the basis of the prespecified inclusion criteria, for example, because they did not explicitly report diagnostic accuracy outcomes), and therefore did not find their way into the current paper.^44^ ^46^ Data in the papers^45^ ^46^ (sensitivities ranging from 5.3% to 14.6% and specificities ranging from 93% to 97%) and from subsequent reviews^9^ indicate that in any case these older studies and scales did not have superior results to those described in our study. Inclusion of these additional scales would not have changed our findings. Although we used a published search strategy,^6^ ^11^ there is a possibility that additional scales were excluded due to the search criteria and of publication bias in the included studies as some studies with negative results may not be widely accessible.

We considered the performance of these scales only in relation to people who self-harmed rather than the wider general or clinical population. However, this is an important clinical group, and in many settings risk scales are an intrinsic part of their management. Our main outcome was repeated self-harm or attempted suicide rather than suicide. While suicide is extremely important, because it is a relatively low-frequency event, it is much harder to predict. This is reflected in the poorer performance of scales in relation to suicide than repeat self-harm as outlined in UK guidance.^6^ Only two of the studies included in this review also reported suicide outcomes.^27^ ^36^ The Manchester Self-Harm Rule identified 100% of the 22 suicide deaths that occurred within the 6-month follow-up period.^27^ The ReACT Rule identified 60 of the 66 suicide deaths (91%) in the derivation data set and 23 of 26 (88%) in the test data set within 6 months of the index episode.^36^ These results indicate high sensitivity, but this is once again at the expense of low specificity and poor positive predictive value. Two other studies combined suicide and repeat self-harm as an outcome,^14^ ^33^ and deaths by suicide were not included in the remaining studies.^15^ ^29^ ^34^ ^35^

### Clinical implications

#### What is the most useful scale following self-harm?

The use of scales is dependent on multiple factors. The scales are not directly comparable due to differences in the incidence of repeat self-harm across studies and methodological quality. Many of the studies were conducted in high-income countries in centrally funded health services,^15^ ^27^ ^33–36^ and so the findings may not be applicable to different settings. The Repeated Episodes of Self-Harm Scale was developed on an inpatient sample which is unlikely to be transferable to emergency department services. The performance of the scales may be additionally influenced by cultural contexts. For example, the Barratt Impulsivity scale^15^ ^40^ was developed in the USA, and the terminology of some of the items may reduce the performance of the scale in other cultures (eg, ‘I squirm at plays or lectures’). There is also a challenging balance when selecting scales based on diagnostic accuracy statistics, and no scale performed well across all indices.

Global indicators such as the diagnostic OR provide the strength of the association between the exposure and the disease and are readily interpreted by clinicians. False-positive and false-negative results are equally weighted, which is advantageous for research and meta-analyses, but may limit clinical use as clinicians cannot evaluate the scale on the basis of sensitivity and specificity.^18^ The scales which had the highest global diagnostic ORs were the Repeated Episodes of Self-Harm scale at the highest threshold (16.34) and the Manchester Self-Harm Rule (10.77).^14^ ^27^

The balance between sensitivity and specificity is dependent on various factors such as resources, purpose of the test and stage of treatment. Clinicians may prefer a test high in sensitivity to capture as many repeat self-harm episodes as possible, for example, the Manchester Self-Harm Rule^27^ or the ReACT Self-Harm rule.^36^ Highly sensitive tests are sometimes used to screen patients or can assist in ‘ruling out’ patients as the possibility of a false negative is relatively low.^19^ The Manchester Self-Harm rule was also validated in other prospective cohort studies and similar sensitivities and specificities were reported.^15^ ^28^ ^35^ ^36^ However, the ReACT Self-Harm Rule^36^ and Manchester Self-Harm Rule^27^ have poor specificity and positive predictive values, and there is a possibility that many patients could be false positives (ie, incorrectly labelled as at high risk), which has cost and resource implications.^47^

Scales high in specificity, such as the Repeated Episodes of Self-Harm scale at the highest threshold,^14^ may be useful for a later stage of assessment or if treatment outcomes are expensive, medically invasive or burdensome to the patients. Scales high in specificity can also be used to ‘rule in’ patients, as the number of false positives is low (so people labelled as at high risk are quite likely to be at high risk). However, the clinical utility of high specificity scales may be limited because of the small numbers of patients who screen positive, and the fact that the high risk of the patients who reach the threshold is already fairly obvious on the basis of conventional clinical risk factors (eg, for the Repeated Episodes of Self-Harm Scale at the highest threshold, the small number of patients who have multiple prior episodes of self-harm, psychiatric diagnosis and recent psychiatric hospitalisation are clearly at elevated risk^14^). The sensitivities of such scales in this study were poor, and there is a possibility of false negatives (people being labelled as at low risk when they are actually at high risk).

Clinicians might consider scales with high positive predictive values such as the Repeated Episodes of Self-Harm scale at the highest threshold, as positive predictive values are a measure of the probability that an individual at high risk actually goes on to repeat self-harm. However, positive predictive values are affected by how common the outcome is, which affects their transferability to clinical settings with a different incidence of repeat self-harm. The scales with high positive predictive values (eg, Repeated Episodes of Self-Harm scale at high threshold,^14^ and Global Severity Index^15^ were also low in sensitivity, which is a further consideration when the evaluating the usefulness of scales for clinical practice.

Scales can be evaluated using likelihood ratios (probability of a specific result among people who repeat self-harm divided by the probability of a given result among people who do not repeat self-harm), and they are widely used in evidence-based medicine.^48^ They are advantageous in evaluating scales, as information from both sensitivity and specificity is used, they are not affected by prevalence, and they are fairly easy to interpret (eg, >10 indicates a useful test). The Repeated Episodes of Self-Harm scale at the highest threshold had the highest likelihood ratio (15.7),^14^ which indicates that the highest risk threshold is useful in predicting repeat self-harm, but had low sensitivity (6%) which limits the scale for screening purposes. There are limitations in the use of likelihood ratios for clinical practice. The estimation of baseline risk may be dependent on clinical experience, accurate estimates of prevalence, and familiarity in expressing risk in terms of probabilities.^49^

Clinicians may prefer to use a scale for predicting completed suicide, but scales which do so are perhaps more likely to have high sensitivity and be over inclusive (eg, the Manchester Self-Harm Rule^27^ and the ReACT Self-Harm Rule.^36^ Only two of the studies in this review^27^ ^36^ evaluated suicide separately as an outcome, and the predictive utility of the scales for suicide needs to be investigated further.

We were unable to examine the predictive usefulness of the scales in predicting shorter versus longer term risk of self-harm repetition due to the heterogeneity of the scales and methodological characteristics. The use of scales in predicting shorter vs longer term risk is clinically important and should be investigated further using prospective cohort studies.

## Conclusion

On the basis of our review, it is clear that no scale appears to perform sufficiently well to be used routinely. The limitations of risk scales in clinical practice are well documented, and it is suggested that the clinical focus should be on ‘conducting comprehensive clinical assessments of each patient’s situation and needs’ rather than the categorisation of patients into high-risk and low-risk categories (p.463).^50–52^ The focus on risk assessment can detract from the therapeutic relationship,^53^ and studies have reported that patients and staff can find assessments with scales an adverse experience.^8^ However, risk scales continue to be widely used in self-harm services with hospitals commonly developing local instruments.^7^ Traditional paradigms which simply aim to balance sensitivity versus specificity may be of limited usefulness in the development of risk scales for use following self-harm. Future research should involve head-to-head comparisons. This may have more validity than comparing scales used in different patient groups across different settings. Studies need to determine the effectiveness of risk scales using robust predictive accuracy cohort studies that are clearly reported according to STARD criteria.^54^ Until then, it is difficult to evaluate what the most useful instruments are and, in line with clinical guidance, scales should not be used in isolation to determine management or to predict risk of future self-harm.^6^

## References

[1] Carroll R, Metcalfe C, Gunnell D Hospital presenting self-harm and risk of fatal and non-fatal repetition: systematic review and meta-analysis. PLoS ONE 2014;9:e89944 10.1371/journal.pone.0089944 24587141 PMC3938547

[2] Owens D, Horrocks J, House A Fatal and non-fatal repetition of self-harm systematic review. Br J Psychiatry 2002;181:193–9. 10.1192/bjp.181.3.193 12204922

[3] Cooper J, Kapur N, Webb R Suicide after deliberate self-harm: a 4-year cohort study. Suicide 2005;162. 10.1176/appi.ajp.162.2.297 15677594

[4] Kapur N, Steeg S, Webb R Does clinical management improve outcomes following self-harm? Results from the multicentre study of self-harm in England. PLoS ONE 2013;8:e70434 10.1371/journal.pone.0070434 23936430 PMC3731259

[5] NICE. Self-harm: the short-term physical and psychological management and secondary prevention of self-harm in primary and secondary care. National clinical guideline number 16. London: NICE, 2004 http://www.nice.org.uk/guidance/cg16 21834185

[6] NICE. Self-harm. The NICE Guideline on Longer-term management. National Clinical Guideline Number 133. NICE Guidelines. London: The British Psychological Society and The Royal College of Psychiatrists, 2011.

[7] Quinlivan L, Cooper J, Steeg S Scales for predicting risk following self-harm: an observational study in 32 hospitals in England. BMJ Open 2014;4:e004732 10.1136/bmjopen-2013-004732 PMC4025469 24793255

[8] Royal College of Psychiatrists. Self-harm, suicide, and risk: helping people who self-harm. Final report of a working group. London: Royal College of Psychiatrists, 2010.

[9] Larkin C, Di Blasi Z, Arensman E Risk factors for repetition of self-harm: a systematic review of prospective hospital-based studies. PLoS ONE 2014;9:e84282 10.1371/journal.pone.0084282 24465400 PMC3896350

[10] Randall JR, Colman I, Rowe BH A systematic review of psychometric assessment of self-harm risk in the emergency department. J Affect Disord 2011;134:348–55. 10.1016/j.jad.2011.05.032 21658779

[11] NICE. Self-harm: Longer-term management. Evidence update April 2013. Evidence update 39. National Collaborating Centre for Mental Health, 2013 https://www.evidence.nhs.uk

[12] Lijmer JG, Mol BW, Heisterkamp S Empirical evidence of design-related bias in studies of diagnostic tests. JAMA 1999;282:1061–6. 10.1001/jama.282.11.1061 10493205

[13] Goldstein RB, Black DW, Nasrallah A The prediction of suicide: sensitivity, specificity, and predictive value of a multivariate model applied to suicide among 1906 patients with affective disorders. Arch Gen Psychiatry 1991;48:418–22. 10.1001/archpsyc.1991.01810290030004 2021294

[14] Spittal MJ, Pirkis J, Miller M The Repeated Episodes of Self-Harm (RESH) score: a tool for predicting risk of future episodes of self-harm by hospital patients. J Affect Disord 2014;161:36–42. 10.1016/j.jad.2014.02.032 24751305

[15] Randall JR, Rowe BH, Colman I Emergency department assessment of self-harm risk using psychometric questionnaires. Can J Psychiatry 2012;57:21. 22296964 10.1177/070674371205700105

[16] Whiting P, Rutjes AW, Reitsma JB The development of QUADAS: a tool for the quality assessment of studies of diagnostic accuracy included in systematic reviews. BMC Med Res Methodol 2003;3:25 10.1186/1471-2288-3-25 14606960 PMC305345

[17] Bossuyt PM, Reitsma JB, Bruns DE The STARD statement for reporting studies of diagnostic accuracy: explanation and elaboration. Ann Intern Med 2003;138:W1–12. 10.7326/0003-4819-138-1-200301070-00012-w1 12513067

[18] Glas AS, Lijmer JG, Prins MH The diagnostic odds ratio: a single indicator of test performance. J Clin Epidemiol 2003;56:1129–35. 10.1016/S0895-4356(03)00177-X 14615004

[19] Sackett DL, Haynes RB, Tugwell P Clinical epidemiology: a basic science for clinical medicine. 2nd edn Lippincott Williams and Wilkins, 1991.

[20] Gerke O, Vach W Number allowed to diagnose. Epidemiology 2014;25:158–9. 10.1097/EDE.0b013e3182a77a81 24296933

[21] Jaeschke R, Guyatt GH, Sackett DL Users’ guides to the medical literature: III. How to Use an article about a diagnostic test B. What are the results and will they help me in caring for my patients? JAMA 1994;271:703–7. 10.1001/jama.1994.03510330081039 8309035

[22] Newcombe RG Two-sided confidence intervals for the single proportion: comparison of seven methods. Stat Med 1998;17:857–72. 10.1002/(SICI)1097-0258(19980430)17:8<857::AID-SIM777>3.0.CO;2-E 9595616

[23] Simel DL, Samsa GP, Matchar DB Likelihood ratios with confidence: sample size estimation for diagnostic test studies. J Clin Epidemiol 1991;44:763–70. 10.1016/0895-4356(91)90128-V 1941027

[24] Armitage P, Berry G Statistical methods in medical research. John Wiley & Sons, 1994

[25] Press W, Teukolsky S, Vetterling W Numerical recipes, 3rd edn. The art of scientific computing: Cambridge University Press, Cambridge, 2007.

[26] RevMan. Review Manager. 5.1 edn Copenhagen: The Cochrane Collaboration, 2011 http://www.cochrane.org/cochrane/revman.htm

[27] Cooper J, Kapur N, Dunning J A clinical tool for assessing risk after self-harm. Ann Emerg Med 2006;48:459–66. 10.1016/j.annemergmed.2006.07.944 16997684

[28] Cooper J, Kapur N, Mackway-Jones K A comparison between clinicians’ assessment and the Manchester Self-Harm Rule: a cohort study. Emerg Med J 2007;24:720–1. 10.1136/emj.2007.048983 17901275 PMC2658442

[29] Carter GL, Clover KA, Bryant JL Can the Edinburgh risk of repetition scale predict repetition of deliberate self-poisoning in an Australian clinical setting? Suicide Life Threat Behav 2002;32:230–9. 10.1521/suli.32.3.230.22175 12374470

[30] Corcoran P, Kelleher MJ, Keeley HS A preliminary statistical model for identifying repeaters of parasuicide. Arch Suicide Res 1997;3:65–74.

[31] Galfalvy HC, Oquendo MA, Mann JJ Evaluation of clinical prognostic models for suicide attempts after a major depressive episode. Acta Psychiatr Scand 2008;117:244–52. 10.1111/j.1600-0447.2008.01162.x 18321353 PMC3773864

[32] Kapur N, Cooper J, Rodway C Predicting the risk of repetition after self harm: cohort study. BMJ 2005;330:394–5. 10.1136/bmj.38337.584225.82 15677364 PMC549109

[33] Waern M, Sjöström N, Marlow T Does the suicide assessment scale predict risk of repetition? A prospective study of suicide attempters at a hospital emergency department. Eur Psychiatry 2010;25:421–6. 10.1016/j.eurpsy.2010.03.014 20620027

[34] Bolton JM, Spiwak R, Sareen J Predicting suicide attempts with the SAD PERSONS scale: a longitudinal analysis. J Clin Psychiatry 2012;73:e735–41. 10.4088/JCP.11m07362 22795212

[35] Bilén K, Ponzer S, Ottosson C Can repetition of deliberate self-harm be predicted? A prospective multicenter study validating clinical decision rules. J Affect Disord 2013;149:253–8. 10.1016/j.jad.2013.01.037 23453675

[36] Steeg S, Kapur N, Webb R The development of a population-level clinical screening tool for self-harm repetition and suicide: the ReACT Self-Harm Rule. Psychol Med 2012;42: 2383–94. 10.1017/S0033291712000347 22394511

[37] Derogatis L BSI brief symptom inventory: administration, scoring, and procedures manual. Minneapolis: National Computer Systems: Inc, 2011.

[38] Patterson WM, Dohn HH, Bird J Evaluation of suicidal patients: the SAD PERSONS scale. Psychosomatics 1983;24:343–5, 348-9. 10.1016/S0033-3182(83)73213-5 6867245

[39] Hockberger RS, Rothstein RJ Assessment of suicide potential by nonpsychiatrists using the SAD PERSONS score. J Emerg Med 1988;6:99–107. 10.1016/0736-4679(88)90147-3 3290325

[40] Patton JH, Stanford MS, Barratt ES Factor structure of the Barratt impulsiveness scale. J Clin Psychol 1995;51:768–74. 10.1002/1097-4679(199511)51:6<768::AID-JCLP2270510607>3.0.CO;2-1 8778124

[41] Skinner HA The drug abuse screening test. Addict Behav 1982;7:363–71. 10.1016/0306-4603(82)90005-3 7183189

[42] Stanley B, Träskman-Bendz L, Stanley M The suicide assessment scale: a scale evaluating change in suicidal behavior. Psychopharmacol Bull 1986;22:200. 3726067

[43] Niméus A, Alsén M, Träskman-Bendz L The suicide assessment scale: an instrument assessing suicide risk of suicide attempters. Eur Psychiatry 2000;15:416–23. 10.1016/S0924-9338(00)00512-5 11112934

[44] Kreitman N, Foster J The construction and selection of predictive scales, with special reference to parasuicide. Br J Psychiatry 1991;159:185–92. 10.1192/bjp.159.2.185 1773234

[45] Hawton K, Fagg J Repetition of attempted suicide: the performance of the Edinburgh predictive scales in patients in Oxford. Arch Suicide Res 1995;1:261–72. 10.1080/13811119508258987

[46] Buglass D, Horton J A scale for predicting subsequent suicidal behaviour. Br J Psychiatry 1974;124:573–8. 10.1192/bjp.124.6.573 4850288

[47] Hatcher S The Manchester Self Harm Rule had good sensitivity but poor specificity for predicting repeat self-harm or suicide. Evid Based Med 2007;12:89 10.1136/ebm.12.5.154 17537897

[48] Phelps MA, Levitt MA Pretest probability estimates: a pitfall to the clinical utility of evidence-based medicine? Acad Emerg Med 2004;11:692–4. 10.1111/j.1553-2712.2004.tb00726.x 15175211

[49] Noguchi Y, Matsui K, Imura H Quantitative evaluation of the diagnostic thinking process in medical students. J Gen Intern Med 2002;17:848–53. 10.1046/j.1525-1497.2002.20139.x PMC1495132 12406355

[50] Ryan CJ, Large MM Suicide risk assessment: where are we now? Med J Aust 2013;198:462–3. 10.5694/mja13.10437 23682873

[51] Large M, Ryan C, Nielssen O The validity and utility of risk assessment for inpatient suicide. Australas Psychiatry 2011;19:507–12. 10.3109/10398562.2011.610505 22077302

[52] Large MM, Nielssen OB Probability and loss: two sides of the risk assessment coin. Psychiatrist 2011;35:413–18. 10.1192/pb.bp.110.033910

[53] Smith MJ, Bouch J, Bradstreet S Health services, suicide, and self-harm: patient distress and system anxiety. Lancet Psychiatry 2015;2:275–80. 10.1016/S2215-0366(15)00051-6 26359905

[54] Moher D, Liberati A, Tetzlaff J Preferred reporting items for systematic reviews and meta-analyses: the PRISMA statement. Ann Intern Med 2009;151:264–9, W64 10.7326/0003-4819-151-4-200908180-00135 19622511

